# Corneal topography in keratoconus evaluated more than 30 years after penetrating keratoplasty: a Fourier harmonic analysis

**DOI:** 10.1038/s41598-020-71818-w

**Published:** 2020-09-10

**Authors:** Takashi Ono, Yuki Kawasaki, Lily Wei Chen, Tetsuya Toyono, Rika Shirakawa, Junko Yoshida, Makoto Aihara, Takashi Miyai

**Affiliations:** Department of Ophthalmology, Graduate School of Medicine, University of Tokyo, 7-3-1 Hongo, Bunkyo-ku, Tokyo, 113-8655 Japan

**Keywords:** Corneal diseases, Medical research

## Abstract

The aim of this observational study was to examine the characteristics of anterior and posterior corneal topography in keratoconic eyes more than 30 years after penetrating keratoplasty (PK). Patients who maintained clear grafts for more than 30 years after PK were included and divided into the keratoconus (KC) group or other diseases (Others) group, based on the primary indication. Twenty-six eyes of 26 patients were included. The KC group and the Others group included 14 eyes and 12 eyes, respectively. The KC group participants were younger at the time of surgery (*P* = 0.03). No differences were found in best-spectacle-corrected visual acuity, keratometric power, and central-corneal-thickness. Based on corneal topography using Fourier harmonic analyses, regular astigmatism in the anterior cornea was significantly larger (*P* = 0.047) and the spherical component in the posterior cornea was significantly lower (*P* = 0.01) in the KC group. The area under the receiver operating characteristic curve of the spherical component, regular astigmatism, asymmetry component, and higher-order irregularity were 66.07%, 63.10%, 57.14%, and 59.23%, respectively, in the anterior cornea and 80.65%, 52.98%, 63.10%, and 63.99%, respectively, in the posterior cornea. Our results suggested that Fourier harmonic analysis of corneal topography could be useful for patients with KC long after PK.

## Introduction

Corneal transplantation is the oldest and most time-proven organ transplantation in the world. Since the first successful operation performed by Eduard Konrad Zirm^1^, many investigators have demonstrated that penetrating keratoplasty (PK) has long-term efficacy and safety^2–4^. In addition, it has been a necessary surgical technique for treating severe corneal diseases. Keratoconus (KC), a corneal disorder that causes progressive corneal protrusion and thinning, is one of the leading indications for PK^5,6^. The clinical outcome after PK for KC is better than that for other diseases such as bullous keratopathy or regrafts^1,7^, and the corneal graft usually survives for a long time. However, KC sometimes recurs and progresses postoperatively, thereby resulting in acute hydrops, reduced visual acuity, and graft failure^8,9^. To understand and detect the recurrence of KC, detailed observation after PK is required. Long-term results of PK have been limited to approximately 15 years, and the outcome data of more than 30 years after PK are scarce^3,4,7,10^.

Corneal topography with Fourier harmonic analysis, which can decompose refractive data into four components (i.e., spherical, regular astigmatism, asymmetry, and higher order irregularity), has been used to investigate corneal irregular astigmatism in various keratoconjunctival diseases^11–13^ and the outcome of surgeries^14–20^, and to detect subclinical KC^21^. The anterior and posterior corneal surfaces can be used effectively and with high sensitivity and specificity to detect differences between a normal cornea and a cornea with subclinical KC^22^. Therefore, observing the anterior and posterior surfaces in patients with KC after PK with corneal topography and Fourier harmonic analysis is clinically important. One study^23^ used this technique to evaluate outcomes 2 years after PK. However, no previous clinical study has conducted Fourier harmonic analysis to assess post-PK results in KC individuals at a longer postoperative time point. The current study aimed to examine the characteristics of corneal topography with Fourier harmonic analysis in KC patients who had received PK more than 30 years earlier.

## Results

Twenty-six eyes of 26 patients were included in the study (16 men and 10 women). The postoperative time after PK was 37.06 ± 5.81 years, and the age at the time of analysis was 67.13 ± 13.92 years (Table 1). The primary diseases treated with PK were as follows: 12 (46.15%) eyes, KC; eight (30.77%) eyes, herpetic keratitis; four (15.38%) eyes, corneal leukoma; one (3.85%) eye, ocular trauma; and one (3.85%) eye, corneal granular dystrophy. Overall, the KC group had 12 eyes and the Others group had 14 eyes.

**Table 1:** Demographic data of patients who maintained clear grafts for more than 30 years after undergoing penetrating keratoplasty.

	All patients	KC group	Others group	*P* value
N	26	12	14	
Sex (men:women)	16:10	10: 2	6: 8	0.05
Age at the operation (years)	30.08 ± 11.94	24.67 ± 6.96	34.71 ± 12.90	0.03*
Postoperative term (years)	37.06 ± 5.81	35.13 ± 4.93	38.72 ± 5.79	0.12
Age at the analysis (years)	67.13 ± 13.92	59.79 ± 6.56	73.41 ± 14.96	0.01*
Best-spectacle-corrected visual acuity (logMAR)	0.28 ± 0.32	0.17 ± 0.32	0.37 ± 0.28	0.11
Intraocular pressure (mmHg)	12.80 ± 3.39	12.88 ± 3.28	12.74 ± 3.36	0.92
Central corneal thickness (µm)	564.65 ± 64.64	540.75 ± 49.18	585.14 ± 66.68	0.07
Thinnest corneal thickness (µm)	500.88 ± 95.50	479.67 ± 96.04	519.07 ± 87.54	0.30
Corneal endothelial cell density (cells/mm^2^)	881.40 ± 419.84	999.71 ± 437.37	777.88 ± 343.69	0.33
Kmax (D)	52.53 ± 7.32	55.28 ± 7.20	50.17 ± 6.25	0.08
AvgK (D)	46.70 ± 5.53	48.81 ± 5.09	44.90 ± 5.04	0.07
Incidence of corneal neovascularization (%)	42.31	58.33	28.57	0.23

All data are expressed as the mean ± the standard deviation.

KC, keratoconus; Others, other corneal diseases; logMAR, logarithm of the minimum angle of resolution; Kmax, maximum keratometric power; AvgK, average keratometric corneal power.

*P < 0.05, based on the unpaired t test.

No significant difference was found between the KC group and the Others group in terms of sex, postoperative time, best spectacle-corrected visual acuity (BSCVA), intraocular pressure (IOP), central corneal thickness (CCT), thinnest corneal thickness (TCT), and endothelial cell density (ECD) (Table 1). However, the age at the time of the operation and age at the time of analysis were significantly lower in the KC group than in the others group (*P* = 0.03 and *P* = 0.01, respectively). No significant difference was found between the two groups in maximum keratometric power (Kmax), average keratometric corneal power (AvgK), and incidence of corneal neovascularization.

In the Fourier harmonic analysis, regular astigmatism of the anterior cornea was significantly larger (*P* = 0.047) and the spherical component of the posterior cornea was significantly lower (*P* = 0.01) for the analysis diameter of 6 mm in the KC group (Table 2). No significant difference existed in any other parameter for the analysis diameter of 6 mm or in all parameters for the analysis diameter of 3 mm.

**Table 2:** Comparison of corneal topography, using Fourier harmonic analysis, between the KC group and Others group.

	All patients	KC group	Others group	*P* value
**Anterior cornea, 3-mm diameter analysis**				
Spherical component (D)	51.59 ± 5.30	53.41 ± 4.14	50.03 ± 5.49	0.11
Regular astigmatism (D)	2.87 ± 2.23	3.66 ± 2.82	2.19 ± 1.05	0.09
Asymmetry component (D)	2.03 ± 1.68	2.02 ± 1.55	2.04 ± 1.73	0.98
Higher order irregularity (D)	0.82 ± 0.58	0.92 ± 0.56	0.74 ± 0.55	0.45
**Anterior cornea, 6-mm diameter analysis**				
Spherical component (D)	51.65 ± 5.71	53.65 ± 4.63	49.94 ± 5.80	0.10
Regular astigmatism (D)	2.75 ± 1.94	3.55 ± 2.38	2.06 ± 0.90	0.047*
Asymmetry component (D)	2.88 ± 2.16	3.04 ± 2.10	2.75 ± 2.13	0.74
Higher order irregularity (D)	0.99 ± 0.64	1.10 ± 0.67	0.90 ± 0.56	0.44
**Posterior cornea, 3-mm diameter analysis**				
Spherical component (D)	− 5.91 ± 2.64	− 5.56 ± 3.73	− 6.21 ± 0.53	0.54
Regular astigmatism (D)	0.54 ± 0.33	0.59 ± 0.38	0.51 ± 0.27	0.55
Asymmetry component (D)	0.48 ± 0.38	0.36 ± 0.26	0.59 ± 0.42	0.13
Higher order irregularity (D)	0.19 ± 0.10	0.16 ± 0.08	0.21 ± 0.10	0.28
**Posterior cornea, 6-mm diameter analysis**				
Spherical component (D)	− 6.36 ± 0.68	− 6.7 ± 0.37	− 6.1 ± 0.73	0.01*
Regular astigmatism (D)	0.50 ± 0.29	0.54 ± 0.31	0.47 ± 0.25	0.59
Asymmetry component (D)	0.61 ± 0.41	0.48 ± 0.27	0.71 ± 0.46	0.15
Higher order irregularity (D)	0.22 ± 0.10	0.19 ± 0.08	0.24 ± 0.11	0.18

All data are expressed as the mean ± the standard deviation.

KC, keratoconus; Others, other corneal diseases.

*P < 0.05, based on the unpaired t test.

For each group, the BSCVA, IOP, CCT, TCT, ECD, Kmax, and AvgK did not significantly change during the 1-year examination period, which occurred more than 30 years postoperatively (Table 3). No significant difference in the 1-year change in BSCVA, IOP, CCT, TCT, ECD, Kmax, and AvgK existed between the two groups (Table 3).

**Table 3:** Comparison of the 1-year changes in characteristics between the KC group and the Others group.

	KC group			Others group			Intergroup *P* value, compared to the value after 1 year	Intergroup *P* value, compared to the 1-year change
	Value after 1 year	One-year change	Intragroup *P* value	Value after 1 year	One-year change	Intragroup *P* value		
Best-spectacle-corrected visual acuity (logMAR)	0.34 ± 0.54	0.16 ± 0.37	0.20	0.43 ± 0.28	0.06 ± 0.31	0.50	0.63	0.49
Intraocular pressure (mmHg)	13.18 ± 3.27	0.67 ± 2.58	0.43	13.36 ± 3.65	0.63 ± 3.3	0.50	0.90	0.97
Central corneal thickness (µm)	536.73 ± 53.12	− 8.27 ± 21.21	0.25	571.93 ± 66.34	− 13.21 ± 25.38	0.08	0.18	0.62
Thinnest corneal thickness (µm)	483.73 ± 95.35	1.00 ± 30.60	0.92	501.14 ± 95.36	− 17.93 ± 39.00	0.12	0.67	0.22
Corneal endothelial cell density (cells/mm^2^)	777.33 ± 188.64	− 145.83 ± 239.35	0.27	840.78 ± 194.10	32.50 ± 242.05	0.78	0.27	0.27
Kmax (D)	58.18 ± 8.62	2.98 ± 4.31	0.05	50.97 ± 6.55	0.80 ± 1.91	0.16	0.03*	0.12
AvgK (D)	48.80 ± 5.40	− 0.17 ± 0.69	0.44	45.10 ± 5.13	0.20 ± 0.66	0.29	0.11	0.20

All data are expressed as the mean ± the standard deviation.

KC, keratoconus; Others, other corneal diseases; logMAR, logarithm of the minimum angle of resolution; Kmax, maximum keratometric power; AvgK, average keratometric corneal power.

*P < 0.05, unpaired t test.

One year after the first evaluation, the spherical component of the posterior cornea was also significantly lower for the analysis diameter of 6 mm in the KC group (*P* = 0.03; Table 4). For each group, Fourier harmonic analysis showed no significant difference in corneal topography during the 1-year evaluation period. The 1-year period change in corneal topography between the two groups was not significantly different.

**Table 4:** Comparison of the 1-year changes in corneal topography, using Fourier harmonic analysis, between the KC group and the Others group.

	KC group			Others group			Intergroup *P* value compared to the value after 1 year	Intergroup *P* value compared to the 1-year change
	Value after 1 year	One-year change	Intragroup *P* value	Value after 1 year	One-year change	Intragroup *P* value		
**Anterior cornea, 3-mm diameter analysis**								
Spherical component (D)	53.50 ± 5.39	0.12 ± 1.76	0.83	50.59 ± 5.42	0.56 ± 0.95	0.05	0.21	0.45
Regular astigmatism (D)	3.85 ± 2.99	− 0.11 ± 1.18	0.78	2.46 ± 1.09	0.27 ± 0.92	0.31	0.14	0.39
Asymmetry component (D)	1.30 ± 0.63	− 0.74 ± 1.46	0.14	1.73 ± 1.70	− 0.31 ± 0.95	0.26	0.45	0.40
Higher order irregularity (D)	0.93 ± 0.68	− 0.03 ± 0.33	0.81	0.71 ± 0.63	− 0.03 ± 0.28	0.72	0.44	0.98
**Anterior cornea, 6-mm diameter analysis**								
Spherical component (D)	53.93 ± 6.15	0.31 ± 1.76	0.59	50.01 ± 5.81	0.06 ± 0.82	0.78	0.13	0.66
Regular astigmatism (D)	3.75 ± 2.56	− 0.05 ± 1.16	0.89	2.16 ± 0.91	0.11 ± 0.69	0.59	0.05	0.69
Asymmetry component (D)	2.77 ± 1.82	− 0.28 ± 0.66	0.20	2.66 ± 2.12	− 0.09 ± 0.75	0.68	0.89	0.52
Higher order irregularity (D)	1.27 ± 0.97	0.12 ± 0.46	0.43	0.97 ± 0.70	0.07 ± 0.27	0.35	0.40	0.76
**Posterior cornea, 3-mm diameter analysis**								
Spherical component (D)	− 6.72 ± 0.40	− 0.04 ± 0.08	0.14	− 5.96 ± 1.69	0.25 ± 1.35	0.51	0.17	0.50
Regular astigmatism (D)	0.61 ± 0.40	− 0.01 ± 0.17	0.80	0.51 ± 0.28	0.00 ± 0.22	0.99	0.50	0.87
Asymmetry component (D)	0.29 ± 0.20	− 0.01 ± 0.06	0.57	0.49 ± 0.33	− 0.10 ± 0.33	0.32	0.11	0.44
Higher order irregularity (D)	0.20 ± 0.16	0.04 ± 0.09	0.26	0.24 ± 0.13	0.04 ± 0.11	0.29	0.57	0.99
**Posterior cornea, 6-mm diameter analysis**								
Spherical component (D)	− 6.72 ± 0.38	− 0.03 ± 0.06	0.21	− 6.18 ± 0.67	− 0.12 ± 0.21	0.06	0.03*	0.18
Regular astigmatism (D)	0.55 ± 0.35	− 0.01 ± 0.15	0.79	0.48 ± 0.24	0.01 ± 0.17	0.88	0.56	0.77
Asymmetry component (D)	0.40 ± 0.20	− 0.04 ± 0.11	0.22	0.62 ± 0.36	− 0.09 ± 0.31	0.31	0.10	0.66
Higher order irregularity (D)	0.23 ± 0.17	0.03 ± 0.10	0.33	0.26 ± 0.14	0.02 ± 0.09	0.40	0.56	0.81

All data are expressed as the mean ± the standard deviation.

KC, keratoconus; Others, other corneal diseases.

*P < 0.05, based on the unpaired t test.

**Figure 1:**
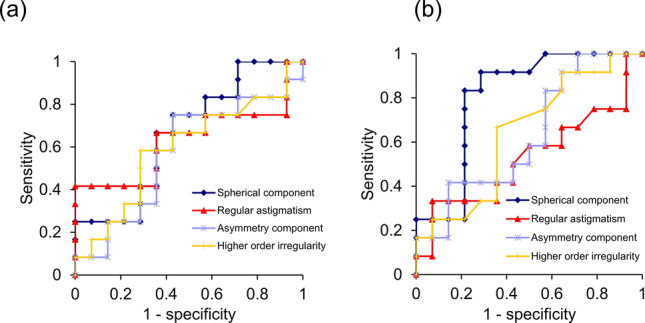
The receiver operating characteristic (ROC) curves of the four components, using Fourier harmonic analysis. (**a**) The ROC curves of the four components of the anterior cornea in the examination with the 6-mm diameter. The areas under the curve (AUCs) of the spherical component, regular astigmatism, asymmetry component, and higher order irregularity are 66.07% (95% confidence interval, 44.38–87.76%), 63.10% (95% confidence interval, 39.08–87.11%), 57.14% (95% confidence interval, 33.59–80.69%), and 59.23% (95% confidence interval, 36.03–82.42%), respectively. (**b**) The ROC curves of the four components, using Fourier harmonic analysis, of the posterior cornea in the examination with the 6-mm diameter. The AUCs of the spherical component, regular astigmatism, asymmetry component, and higher order irregularity are 80.65% (95% confidence interval, 62.59–98.72%), 52.98% (95% confidence interval, 28.92–77.03%), 63.10% (95% confidence interval, 40.80–85.39%), and 63.99% (95% confidence interval, 41.91–86.06%), respectively.

To demonstrate the accuracy in detecting the difference between the KC group and the Others group, a receiver operating characteristic (ROC) curve was examined in the four components of Fourier harmonic analysis in the cornea with the diameter of 6 mm (Fig. 1a,b). For the analysis of the anterior cornea, the area under the curve (AUC) values of the spherical component, regular astigmatism, asymmetry component, and higher order irregularity were 66.07% (95% confidence interval, 44.38–87.76%), 63.10% (95% confidence interval, 39.08–87.11%), 57.14% (95% confidence interval, 33.59–80.69%), and 59.23% (95% confidence interval, 36.03–82.42%), respectively. The cut-off points were 50.29 D, 4.58 D, 1.61 D, and 0.86 D, respectively, and the Youden indices were 0.32, 0.42, 0.32, and 0.30, respectively. For the analysis of the posterior cornea, the AUCs of the four components were 80.65% (95% confidence interval, 62.59–98.72%), 52.98% (95% confidence interval, 28.92–77.03%), 63.10% (95% confidence interval, 40.80–85.39%), and 63.99% (95% confidence interval, 41.91–86.06%), respectively. The cut-off points were − 6.37 D, 0.86 D, 0.98 D, and 0.19 D, respectively, and their Youden indices were 0.63, 0.26, 0.29, and 0.31, respectively. The AUC of the spherical component of the posterior cornea in the analysis of the 6 mm diameter was significantly larger than 0.5 (*P* < 0.001), although the AUCs of the other parameters did not significantly differ from 0.5.

## Discussion

This clinical study found that KC was the most frequent (46.15%) primary disease for grafts that survived for more than 30 years after PK. This finding seemed to corroborate the results of Thompson et al.^2^, who reported that eyes with a preoperative diagnosis of KC had the highest graft survival rates for the 5-year outcome (97%) and the 10-year outcome (92%). The mean age at the time of surgery of our patients was 30.08 ± 11.94 years, which was relatively low for patients who underwent PK. In particular, the mean age at the time of surgery in the KC group (24.67 ± 6.96 years) was significantly lower than that in the Others group (34.71 ± 12.90 years). An older age in a recipient is one risk factor for graft failure^24^; therefore, the corneal grafts of patients who were young when they underwent PK could have survived longer. In addition, many patients who underwent PK at an older age were possibly too old to visit a hospital more than 30 years later.

The corneal shape can gradually change after PK for KC, and several studies have investigated the parameters that could be useful for its assessment. Yoshida et al.^8^ previously reported that a large corneal cylinder power was a risk factor for recurrent KC after PK. We analyzed corneal topography in more detail and demonstrated that regular astigmatism in the anterior cornea was significantly larger in the KC group than in the Others group; this result suggested the progression of recurrent keratoconic changes in the KC group. In addition, Oshika et al.^25^ analyzed preoperative and postoperative data by using Fourier harmonic analysis and found that all four parameters (i.e., spherical component, regular astigmatism, asymmetry component, and higher order irregularity) in the anterior cornea were high in KC eyes. Although our study included only patients after PK and was not directly comparable to that of Oshika et al.^25^, we also found that the spherical component, asymmetry component, and higher order irregularity in the anterior cornea tended to be larger in the KC group. Hayashi et al.^23^ longitudinally examined the changes that occurred in the corneal surface 2 years after PK and reported that corneal surface configuration becomes stable after 6 months. Our data newly suggested that, based on an extended long-term perspective, corneal topographic changes can occur gradually after PK for KC, compared to PK for other diseases.

Compared to the anterior cornea, posterior corneal changes occur earlier in any ectatic disease and require careful observation^5^. In our analysis of the posterior cornea, the spherical component was significantly lower in the KC group than in the Others group. A significant difference was also confirmed with data obtained during the 1-year evaluation period (Table 4). These findings indicated that ectatic changes occurred in patients in the KC group.

In our study, posterior keratometric data were examined to a greater extent, compared to previous research. Sideroudi et al.^22^ reported that regular astigmatism, the asymmetry component, and higher order irregularity in Fourier harmonic analysis were significantly different between healthy eyes and eyes with subclinical KC or KC; however, they did not fully examine postoperative topographic changes in the posterior cornea. In the current study, we investigated parameters in the posterior cornea that distinguished the KC group from the Others group, based on Fourier harmonic analysis. The results of the ROC curves showed that the AUC of the spherical component of the posterior cornea in the analysis of the 6 mm diameter was significantly larger than 0.5 (*P* < 0.001). This finding suggested that the analyses of the spherical component of the posterior cornea can provide useful information for detecting ectatic changes of KC patients long after PK.

Different tools can facilitate the early diagnosis of subclinical KC, which is clinically important for careful observation^26–28^. Steinberg et al.^29^ reported that anterior segment optical coherence tomography (AS-OCT) can be used to discriminate between normal eyes and subclinical KC eyes. In addition, clinicians are required to detect the recurrence and progression of KC in patients after PK; however, the detection of corneal topographic changes with software is not always possible because of wound sutures or eccentricity. Our data suggested that corneal topographic changes progressed in patients with KC in the 30 years following PK, although a significant 1-year change in topography was not detected. Our results add support to the accuracy of AS-OCT and the utilization of AS-OCT, compared to the accuracy and utilization of older tools such as the Topographic Modeling System (Tomey Corporation, Nagoya, Japan)^25,30^ and the Scheimpflug imaging system^21,22^, which are also useful for KC. Moreover, differences in components using Fourier harmonic analysis were detected for the analysis of the 6-mm diameter, but not for the analysis of the 3-mm diameter. Given that topographic changes after PK in patients with KC possibly occur at the pericenter or inferior side of the cornea rather than at the center^8^, our research also suggested that AS-OCT analysis with a wider area can more sensitively detect changes in the cornea and be a useful evaluation tool. In addition, this approach using AS-OCT with Fourier harmonic analysis could be applicable for the long-term evaluation of other procedures involving corneal transplantation for KC such as deep anterior lamellar keratoplasty (DALK). However, we did not include patients who had undergone DALK more than 30 years earlier because, at that time, the DALK technique had just been invented.

The current study has several limitations. First, the study design was retrospective, and the number of patients was small owing to the rarity of patients who have maintained clear grafts for more than 30 years after PK^31^. Our results successfully disclosed significant differences in some parameters with Fourier harmonic analysis, although the small patient number could have resulted in a low detection power. Second, the frequency at which corneal topographic analysis was conducted was limited. With relatively stable corneal surfaces, patients did not need to frequently visit a medical facility and undergo corneal topographic analysis. Third, some patients underwent PK at another institution and their data were unavailable for preoperative and postoperative comparison. Prospective clinical studies that examine more items, compare the preoperative and postoperative data, and detect risk factors for recurrence are required to overcome these limitations.

In conclusion, we retrospectively examined the characteristics of corneal topography more than 30 years after PK by using Fourier harmonic analysis. In the KC group, regular astigmatism in the anterior cornea was significantly larger and the spherical component in the posterior cornea was significantly smaller than those in the Others group. This finding suggested that corneal topographic analysis using Fourier harmonic analysis is useful for detecting topographic differences in patients with KC and in patients with other diseases long after having undergone PK. To evaluate time-dependent changes in corneal topography in detail, further clinical study with more cases is required.

## Methods

This retrospective observational clinical study was approved by the Institutional Review Board of the Research Ethics Committee of the University of Tokyo Hospital (Tokyo, Japan) and written informed consent was waived with providing participants the opportunity to opt out from the study. This study also adhered to the tenets of the Declaration of Helsinki. The study included patients who had visited the Cornea Clinic of the University of Tokyo Hospital between December 2015 and December 2018 and had maintained clear grafts for more than 30 years after PK. The data of the first visit day were selected if the data were acquired multiple times during the examination periods. We selected one eye from each patient; we included the eye in which the AS-OCT topological data were more accurately acquired when the patients had undergone PK for both eyes. Patients who were not evaluated with AS-OCT at the observation period or patients whose medical charts provided insufficient information were excluded from the study. The patients were divided into two groups, based on the primary indication for PK: the KC group and the Others group.

The BSCVA, IOP, ECD, CCT, TCT, Kmax, AvgK, and corneal topography evaluated by using Fourier harmonic analyses of the topographic data were reviewed from the patients’ medical records and compared between the two groups. In addition, the values 1 year after the procedure were also reviewed and the 1-year changes were compared between the two groups. The BSCVA was measured with decimal visual acuity and was converted to the logarithm of the minimum angle of resolution (logMAR). The IOP was measured with applanation tonometry. The CCT, TCT, Kmax, AvgK, and corneal topography were examined using AS-OCT (CASIA or CASIA2; Tomey, Tokyo, Japan). For poor scan images, the examiners digitally elevated the patients’ upper eyelid while paying careful attention to corneal deformation. Fourier harmonic analysis of corneal topographic data was conducted, as previously reported^15^. Axial refractive power data on the mire ring were decomposed with a series of trigonometric components, as follows. Dioptric powers on mire ring i, Fi(σ), were transformed into the trigonometric components of the form with the Fourier series harmonic analysis program included in CASIA or CASIA2 software (Tomey Corporation):

$${\text{F}}_{{\text{i}}} \left( \sigma \right) = a_{0} + c_{1} \cos \left( {\sigma - \alpha_{1} } \right) + c_{2} \cos 2\left( {\sigma - \alpha_{2} } \right) + c_{3} \cos 3\left( {\sigma - \alpha_{3} } \right) + \cdots + c_{n} \cos n\left( {\sigma - \alpha_{n} } \right)$$

in which *a0* is the spherical component of the ring, *2c1* is the asymmetry component (i.e., tilt or decentration), *2c2* is regular astigmatism, and the summation of *c*_*3*_ … *c*_*n*_ includes the higher order irregularity components. They were averaged for each parameter (i.e., spherical component, regular astigmatism, asymmetry component, and higher order irregularity). All calculations were conducted using CASIA or CASIA2 (Tomey Corporation). ROC curves were applied to determine the predictive accuracy of the examination for the four parameters of the Fourier harmonic analysis. The ROC curve was expressed by plotting with sensitivity and 1—specificity. The AUC, cut-off points, and Youden index were calculated.

All data are expressed as the mean ± standard deviation, unless otherwise mentioned. The unpaired t-test was used for intergroup comparison for the continuous variables. The paired t-test was used for intragroup comparison for the 1-year change. The chi-square test was used for categorical variables and the AUC comparison, and Fisher’s exact t-test was used for comparisons of inadequate sample sizes. Two-tailed *P* value < 0.05 was statistically significant. Statistical analyses were conducted using the BellCurve for Excel (Social Survey Research Information, Tokyo, Japan).

## Data Availability

The data that support the findings of this study are available on request from the corresponding author (T.M.). The data are not publicly available because they contain information that could compromise the privacy of the research participants.
